# Investigation of the Mechanisms of Chuankezhi Injection in the Treatment of Asthma Based on the Network Pharmacology Approach

**DOI:** 10.1155/2021/5517041

**Published:** 2021-06-10

**Authors:** Hao Zhu, Yuhuan Shi, Shanshan Jiang, Xiuxiu Jiao, Hui Zhu, Rong Wang, Yongfang Yuan

**Affiliations:** Department of Pharmacy, Shanghai Ninth People's Hospital, Shanghai Jiao Tong University School of Medicine, Shanghai, China

## Abstract

**Background:**

Chuankezhi injection (CKZI) was an effective traditional Chinese medicine (TCM) injection in adjuvant bronchial asthma therapy. In this report, we used a network pharmacology method to reveal the mechanisms of CKZI for the treatment of asthma.

**Methods:**

The candidate compounds in CKZI were determined by searching the Traditional Chinese Medicine Systems Pharmacology Database and Analysis Platform (TCMSP) and China National Knowledge Infrastructure website (CNKI). The targets of candidate compounds were searched in the TCMSP, DrugBank 5.0, and SwissTargetPrediction. The disease targets were screened from the Online Mendelian Inheritance in Man (OMIM) and GeneCards. The overlapping gene symbols between candidate compounds and disease were filtered via a Venn diagram and were considered as potential targets. A protein-protein interaction (PPI) network and disease-related candidate compound-target-pathway (DC-T-P) network were visualized by Cytoscape 3.6.1. Gene Ontology (GO) functions and Kyoto Encyclopedia of Genes and Genomes (KEGG) pathway enrichment analysis were performed by metascape to determine the pathways related to asthma.

**Results:**

A total of 70 overlapping gene symbols were recognized as potential targets. Cytokines (IL6, TNF, and IL1B) and chemokines (CXCL8 and CCL2) could be recognized as hub genes. Asthma-related candidate compounds were mainly flavonoids, such as quercetin, luteolin, and kaempferol. The cytokine-mediated signaling pathway, cytokine receptor binding, and membrane craft were the most significant biological process (BP), molecular function (MF), and cellular component (CC) of GO function results, respectively. The relevant pathways of CKZI against asthma mainly include IL-17, NF-kappa B, HIF-1, calcium, and PI3K-Akt signaling pathways.

**Conclusion:**

Our research provided a theoretical basis for further investigating the mechanisms of CKZI in the treatment of asthma.

## 1. Introduction

Bronchial asthma was reported as the most prevalent chronic inflammatory airway disease, and it affected 1–18% of the global population [1]. Unfortunately, a large number of Chinese people suffered from asthma were undiagnosed and undertreated [2, 3]. Asthma not only caused cough and short of breath but also led to respiratory failure and ultimately endangered human health. The universally acknowledged pathological processes of asthma mainly included chronic airway inflammation, airway hyperresponsiveness, reversible airway restriction, and airway remodeling due to airway immune inflammation and neuromodulation. [4–6]

Up to now, the combination of inhaled corticosteroid (ICS) and long-acting *β*-receptor agonist (LABA) (e.g., budesonide and formoterol) remained the first-line therapy in the treatment of chronic asthma. Combination therapy could prevent and reduce the rate of exacerbations compared with ICS treatment alone [5]. It could also reduce the dosage of ICS, thus improving the compliance of patients and reducing side effects. Other medications for chronic asthma patients included antileukotrienes, tiotropium, inhaled or nebulized short-acting *β*2-agonist (SABA), ipratropium bromide, environmental control, and targeted biologic therapy, such as anti-IgE (omalizumab) and anti-IL-5 [7]. Nevertheless, some patients had poor compliance because they could not tolerate adverse reactions of ICS and other drugs, leading to deterioration of asthma. Besides, severe asthma patients could be hardly controlled even with adequate medications. Thus, it is urgent to find safe and effective medications in the treatment of asthma.

Traditional Chinese Medicine (TCM), featured as having “multiple ingredients, multiple targets, and multiple pathways,” has been used for treating complex systemic diseases for thousands of years in China. TCM attracted scientists' attention as it had unique advantages, such as fewer side effects, attenuated symptoms, and life quality improvement [8, 9]. Chuankezhi injection (CKZI, TCM injection) was approved by the NMPA for the treatment of bronchial asthma. It contained two main Chinese herbs, Yingyanghuo (YYH, dried leaves of *Epimedium brevicornu* Maxim., *Epimedium sagittatum* (Siebold and Zucc.) Maxim., *Epimedium pubescens* Maxim., and *Epimedium koreanum* Nakai) and Bajitian (BJT, *Morinda officinalis* F.C.How). Several researches have shown that CKZI could relieve cough and asthma, exerting antiallergy, anti-inflammatory, stress response, and immunoregulatory functions. CKZI might adjust the imbalance of Th1/Th2 and reduce the expression level of IL-17 of asthma patients [10–14]. However, the explicit mechanisms of CKZI for asthma treatment have not been fully elucidated.

Network pharmacology has emerged as an effective approach to explore the internal mechanisms of multicomponent drugs. Based on the database information of genomics, proteomics, diseases, and drugs, we could construct the visualized interaction network of “drug-component-target-pathway-disease” (D-C-T-P-D) to investigate the therapeutic effects of active ingredients and targets on the network. Network pharmacology created the “multicomponent” and multitarget” mode and has been used to reveal the potential mechanisms of compound Chinese herbal extracts or preparations [15–17].

In this report, we used a network pharmacology method to investigate the therapeutic effects of CKZI in the treatment of asthma. We screened the candidate compounds in CKZI and their corresponding targets. The overlapping targets of asthma and candidate compounds were analyzed, and a disease-related candidate compound-target-pathway (DC-T-P) network was constructed.

## 2. Materials and Methods

### 2.1. Candidate Compounds Screening and Related Target Identification

Firstly, candidate compounds in YYH and BJT were screened in the Traditional Chinese Medicine Systems Pharmacology Database and Analysis Platform (TCMSP, http://tcmspw.com/tcmsp.php/) [18] and China National Knowledge Infrastructure website (CNKI, https://www.cnki.net/). Secondly, drug-likeness (DL)≥0.18 and oral bioavailability (OB)≥30% were set as the thresholds to select candidate compounds via ADME (absorption, distribution, metabolism, and excretion) analysis. Thirdly, we used TCMSP, DrugBank 5.0 (https://www.drugbank.ca/) [19] and SwissTargetPrediction (http://www.swisstargetprediction.ch/) [20] to screen the related targets of candidate compounds. Finally, all the target names were transferred to standard gene symbols by UniProt (http://www.UniProt.org/). The species was selected as “*Homo sapiens* (Human),” and nonhuman gene symbols were eliminated.

### 2.2. Herb-Candidate Compound-Target (H–C-T) Network Construction

Candidate compounds in YYH and BJT and the screened targets (gene symbols) were input into the Cytoscape 3.6.1 software to construct a Herb-Candidate Compound-Target network. A graphical network represents the connection and interaction between target genes and compounds, and the topological properties of the network were analyzed through Network Analyzer (default setting). The degree value of a node (candidate compound or target) indicates the number of connections between the node and other nodes. Specifically, the greater the degree value is, the more critical the node is.

### 2.3. Disease Targets and Potential Target Identification

Bronchial-asthma-related targets were searched from the Online Mendelian Inheritance in Man (OMIM; https://omim.org/) and GeneCards (https://www.genecards.org/) [21] databases. The search keywords of the related disease were restricted to “bronchial asthma” and “asthma.” Furthermore, the species was set to “*Homo sapiens* (Human).” All the targets were normalized to standard gene symbols by UniProt (http://www.UniProt.org/), and duplications were removed. The overlapping gene symbols between candidate compounds and disease were filtered via a Venn diagram and were considered as potential targets of CKZI in the treatment of asthma.

### 2.4. Protein-Protein Interaction (PPI) Network Construction

PPI is the process by which two or more proteins form a protein complex through noncovalent bonds. The PPI network was constructed by importing potential targets into the STRING 11.0 database (https://string-db.org/) [22, 23] and analyzed. “*Homo sapiens* (Human)” was chosen, and a scoring value > 0.7 was selected as a high confidence basis for protein interactions. PPI network files were imported into Cytoscape 3.6.1 software to construct the PPI network, and the topological properties of the PPI network were analyzed through Network Analyzer (default settings).

### 2.5. GO Functions and KEGG Pathway Enrichment Analysis

To reveal the potential biological function of CKZI in the treatment of asthma, Gene Ontology (GO) functions and Kyoto Encyclopedia of Genes and Genomes (KEGG) pathway enrichment analysis were performed by metascape (https://metascape.org/) [24]. GO terms were grouped into the following three categories: the biological process (BP), molecular function (MF), and cellular component (CC). *P* value < 0.01, minimum overlap≥3, and minimum enrichment≥1.5 were considered as the cutoff criteria.

### 2.6. Disease-Related Candidate Compound-Target-Pathway (DC-T-P) Network Construction

Disease-related targets were selected by analyzing KEGG pathway enrichment, and disease-related candidate compounds in two herbs were screened by comparing with disease-related targets. Candidate compounds that did not contain disease-related targets were eliminated. To analyze the association among the disease-related potential targets, candidate compounds, and pathways, a DC-T-P network was constructed using Cytoscape 3.6.1 software, and the topological properties of the network were analyzed through Network Analyzer (default settings).

## 3. Results

### 3.1. Candidate Compound Screening and Related Target Identification

**Figure 1:**
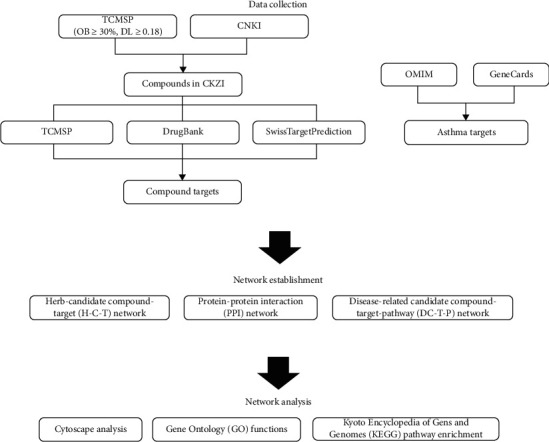
Scheme of the network pharmacology analysis process.

Figure 1 illustrate the analysis process of the network pharmacology method. We first collected the data of compound targets in CKZI and asthma-related targets. Then, the network was established and analyzed by Cytoscape software. After screening in TCMSP and filtrating by DL ≥ 0.18 and OB ≥ 30%, 23 compounds in YYH and 20 compounds in BJT met the criteria. However, some key components in these two herbs were not included. After searching the literature in CNKI, 5 compounds (sagittatoside A, sagittatoside B, icariside I, icariside II, and chlorogenic acid) in YYH and 5 compounds (2-hydroxy-3-methylanthraquinone, 1,6-dihydroxy-2,4-dimethoxyanthraquinone, 1-methoxy-2-hydroxyanthraquinone, deacetyl asperulosidic acid, and monotropein) in BJT were added. Next, we used TCMSP, DrugBank 5.0, and SwissTargetPrediction to predict the related targets of compounds. The probability of the targets searched in SwissTargetPrediction was set as beyond 0.9. After removing duplication and compounds without any targets (2 compounds in YYH and 6 compounds in BJT), 26 compounds in YYH and 19 compounds in BJT were considered as candidate compounds in CKZI. All the target names were transferred to standard gene symbols by UniProt. The chemical structures and profiles of total 45 candidate compounds are shown in Table S1.

### 3.2. Herb-Candidate Compound-Target (H–C-T) Network Construction

**Figure 2:**
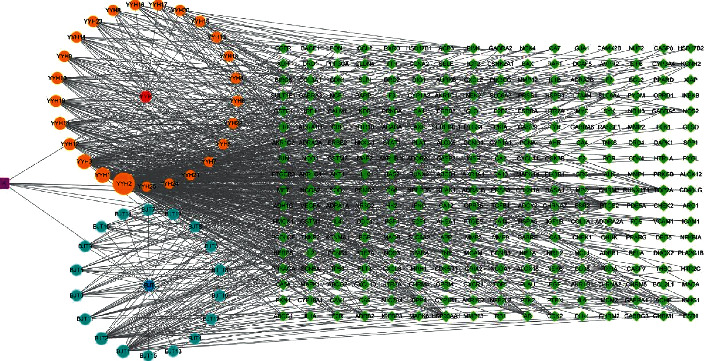
Herb-candidate compound-target (H–C-T) network (YYH: Yingyanghuo; BJT: Bajitian; a overlapped compound in YYH and BJT).

The candidate compounds selected from YYH were mainly flavonoids, sterols, alkaloids, esters, and phenolic acids, and compounds selected from BJT were mainly anthraquinones, sterols, esters, and flavonoids. To analyze the association among herbs, candidate compounds, and related targets, we then constructed the herb-candidate compound-target (H–C-T) network by using Cytoscape 3.6.1 software. A graphical network is shown in Figure 2, and the nodes of different colors and shapes represent different herbs, candidate compounds, and related targets. The degree value of compounds and targets is shown in Tables S2 and S3 separately. The nodes and edges of the network were 334 and 874, respectively. The degree value of the candidate compounds was between 2 and 205, and the degree of the potential targets ranged from 1 to 30. The greater the degree value, the bigger the node as shown in Figure 2. Among the candidate compounds, quercetin (YYH2) exhibited the greatest degree value, 205. 10 candidate compounds showed a degree value beyond 20, and they were quercetin (YYH2), luteolin (YYH1), kaempferol (YYH3), anhydroicaritin (YYH12), 2,7-dihydrohomoerysotrine (YYH14), beta-sitosterol (BJT1), 2-hydroxy-3-methylanthraquinone (BJT2), 8-prenyl-flavone (YYH19), 8-isopentenyl-kaempferol (YYH10), and chryseriol (YYH9). A total of 288 gene symbols were included in the analysis, and PTGS2 showed the greatest degree value, 30. Top 10 gene symbols were PTGS2, NCOA2, HSP90AA1, PTGS1, TOP2A, SCN5A, PIK3CG, CALM1, PRSS1, and F10, respectively.

### 3.3. Disease Target and Potential Target Identification

**Figure 3:**
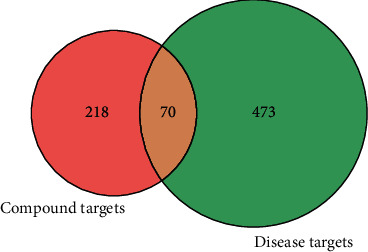
Venn diagram of overlapping gene symbols.

Bronchial-asthma-related targets were searched from OMIM and GeneCards databases. The relevance score in GeneCards was set as 6.125. After normalized to standard gene symbols by UniProt and removing duplicates, 543 gene symbols were selected as disease-related targets. The potential targets of CKZI in treating asthma were considered as the overlapping gene symbols between candidate compounds and disease. The result is shown in Figure 3, and 70 overlapping gene symbols were recognized as potential targets.

### 3.4. Protein-Protein Interaction (PPI) Network Construction

**Figure 4:**
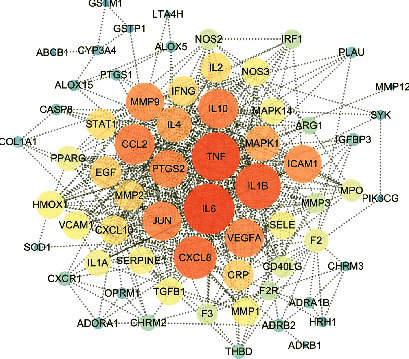
Potential target protein-protein interaction (PPI) network.

Based on 70 potential targets, a PPI network was established by importing the standardized gene symbols of the potential targets into the STRING 11.0 database. Cytoscape 3.6.1 software was used to visualize the PPI network. As illustrated in Figure 4, the color and size of the nodes reflected the degree value, and larger and darker nodes meant a greater degree value. The PPI network contained 65 nodes and 445 edges after selecting the scoring value > 0.7 as a high confidence basis for protein interactions. The degree values of 65 gene symbols are listed in Table S4, and among them, 8 gene symbols showed the degree value ≥ 28. They were IL6 (degree = 40), TNF (degree = 38), IL1B (degree = 35), CXCL8 (degree = 32), CCL2 (degree = 29), IL10 (degree = 29), VEGFA (degree = 28), and JUN (degree = 28).

### 3.5. GO Pathway Enrichment Analysis

**Figure 5:**
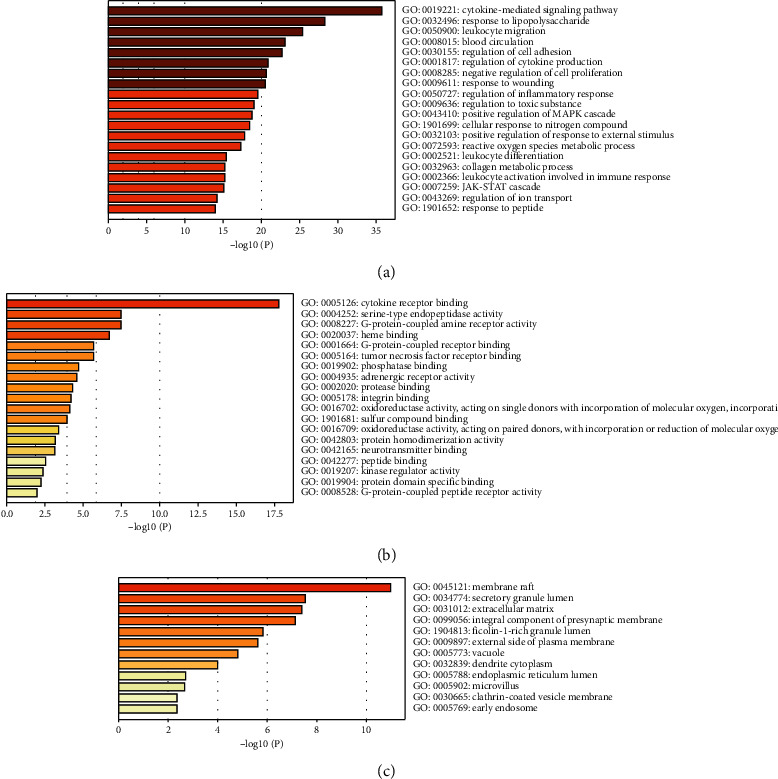
GO enrichment analysis. Biological process (a), molecular function (b), and cellular component (c).

GO (Gene Ontology) enrichment analysis was annotated from three aspects: the biological process (BP), molecular function (MF), and cellular component (CC). To reveal the pharmacological mechanisms of CKZI in asthma, 70 potential targets were put into the metascape database for annotation GO enrichment analysis and a *P* value < 0.01 was considered as the cutoff criterion.  Figure 5(a) shows the top 20 BP enrichment results. Figures 5(b) and 5(c) show all MF and CC enrichment results. The cytokine-mediated signaling pathway, cytokine receptor binding, and membrane craft were the most important BP, MF, and CC of CKZI against asthma. Cytokine-mediated signaling pathways, response to lipopolysaccharide, leukocyte migration, blood circulation, and regulation of cell adhesion were the top 5 BP of CKZI against asthma.

### 3.6. KEGG Pathway Enrichment Analysis

**Figure 6:**
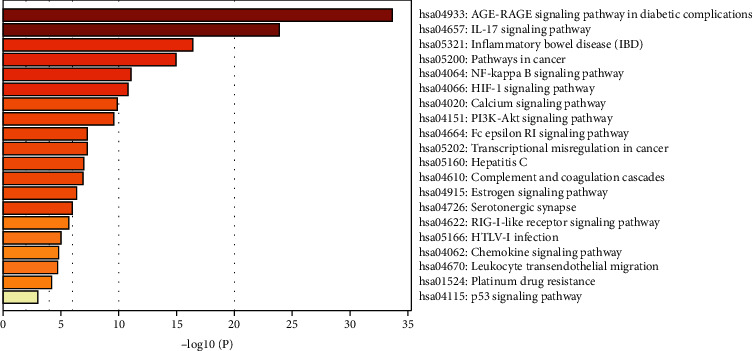
KEGG pathway enrichment analysis.

KEGG (Kyoto Encyclopedia of Genes and Genomes) pathway enrichment analysis of the 70 potential targets was conducted using the Metascape database. Based on the criterion of a *P* value < 0.01, a total of 22 pathways were obtained and the top 20 pathways were shown as the core pathways, as shown in Figure 6. The results indicated that relevant pathways of CKZI against asthma mainly include the IL-17 signaling pathway, NF-kappa B signaling pathway, HIF-1 signaling pathway, calcium signaling pathway, and PI3K-Akt signaling pathway.

### 3.7. Disease-Related Candidate Compound-Target-Pathway (DC-T-P) Network Construction

**Figure 7:**
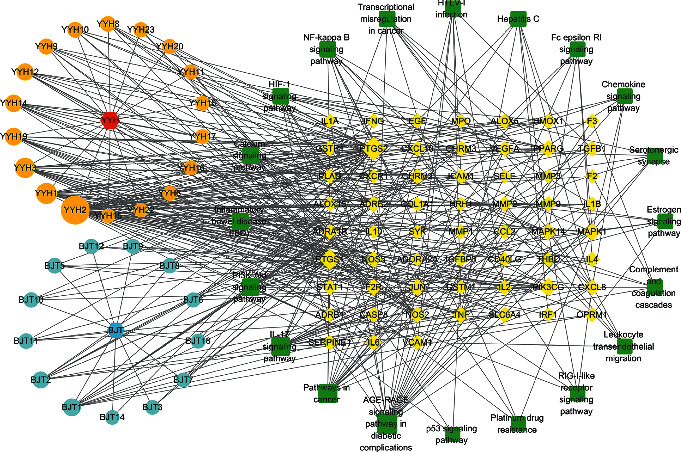
Disease-related candidate compound-target-pathway (DC-T-P) network (YYH: Yingyanghuo; BJT: Bajitian).

Asthma-related candidate compounds, targets, and pathways were screened by the results of KEGG pathway enrichment analysis. A graphical network was constructed by Cytoscape 3.6.1 and is shown in Figure 7. Nodes of different colors and shapes represented different compounds, targets, and pathways, while lines represented the interactions between them. After analysis, 18 compounds in YYH and 13 in BJT were considered as disease-related candidate compounds. A total of 59 targets related to 20 pathways were selected. The degree values of compounds, targets, and pathways are listed in Tables S5, S6, and S7, respectively. The greater the degree value, the bigger the node, as shown in Figure 7. The top 5 candidate compounds were quercetin, luteolin, kaempferol, 8-prenyl-flavone, and beta-sitosterol, and the top 5 pathways related to asthma were the IL-17 signaling pathway, NF-kappa B signaling pathway, HIF-1 signaling pathway, calcium signaling pathway, and PI3K-Akt signaling pathway.

## 4. Discussion

Bronchial asthma was the most prevalent chronic inflammatory airway disease, and the first-line treatment was combination treatment of ICS and LABA. However, severe side effects of ICS and LABA and poor compliance of severe asthma patients limited their applications in clinic. CKZI was a compound TCM injection that mainly included two Chinese herbal extracts, Yingyanghuo (dried leaves of *Epimedium brevicornu* Maxim., *Epimedium sagittatum* (Siebold and Zucc.) Maxim., *Epimedium pubescens* Maxim., and *Epimedium koreanum* Nakai) and Bajitian (*Morinda officinalis* F.C. How). CKZI was widely utilized in adjuvant therapy of asthma, but its mechanisms have not been systematically and comprehensively elucidated. Herein, we used a network pharmacology method to construct a disease-related candidate compound-target-pathway (DC-T-P) network to visualize the connection between CKZI and asthma.

After screening and analysis, quercetin, luteolin, and kaempferol were regarded as the most important compounds extracted from CKZI for asthma treatment. The three compounds were all flavonoids. Flavonoids possessed anti-inflammatory, antioxidant, and antiallergic, as well as immune-modulating, effects. Numerous *in vitro* and *in vivo* studies have found that flavonoids had the potential to inhibit the onset and development of inflammatory diseases, including asthma [25, 26].

70 overlapping gene symbols between candidate compounds and asthma were recognized as potential targets. From PPI analysis results, cytokines such as IL6, TNF, IL1B, and IL10 and chemokines such as CXCL8 and CCL2 could be recognized as hub genes.

The research showed that TH1/TH2 imbalance, cytokines, matrix metalloproteinase, tissue inhibitor of metalloproteinase, genetic factors, and neural regulation all played essential roles in the occurrence and progression of the chronic airway inflammatory process in asthma [27–30].

Among the abovementioned physiological processes, cytokines and chemokines were widely investigated in the pathogenesis of asthma. Study has found that asthma was more severe in patients with excessive cytokine expression, such as IL6, IL1B, and TNF-*α*. Typically, IL6 is regulated by IL1B, and IL1B could cause airway epithelial cell dysfunction. Another research showed that higher levels of IL-6 and MMP-9 were observed in asthma patients compared to the control group and in severe asthma as compared to moderate asthma [31]. Tumor necrosis factor-*α* (TNF-*α*), an important cytokine in the innate immune response, can incite inflammatory response and promote airway epithelium injury and airway hyperresponsiveness. It could also stimulate the induction of inflammatory cytokines, including IL1B, IL6, and IL8 [28, 32]. Chemokines (CXCL8) and their cognate receptors were also important stimulators in the immune system by mediating the activation and trafficking of immune cells during innate and adaptive responses. [33].

We performed GO function and KEGG pathway enrichment to reveal the related signaling pathway as cluster analysis could exhibit a refined detail of CKZI for treating asthma compared to common compound-target analysis. The cytokine-mediated signaling pathway, cytokine receptor binding, and membrane craft were the most significant biological process, molecular function, and cellular component of GO function results, respectively. The result was in accordance with hub genes selected by PPI analysis, indicating cytokines and chemokines were important biomarkers in the progression of asthma.

The IL-17 signaling pathway was the most important pathway in the treatment of asthma by CKZI. IL-17 A was associated with neutrophilic inflammation and corticosteroid insensitivity in different asthma conditions, including allergic asthma, severe asthma, and asthma exacerbations. In addition, IL-17A has been shown to decrease the responsiveness of CXCL-8 production by the bronchial epithelium to corticosteroids. In the majority of asthma patients, primary bronchial epithelial cells were hyperresponsive to IL-17A and proinflammatory stimuli such as TNF-*α*, reflected by an enhanced production of inflammatory mediators such as CXCL-8, and corticosteroid insensitivity [34–36]. Other signaling pathways associated with airway inflammation such as NF-*κ*B, PI3K-Akt, and HIF-1 were also relatively important pathways.

## 5. Conclusions

Network pharmacology was an effective approach to explore the internal mechanisms of multicomponent drugs, particularly TCMs. After analysis, we found that flavonoids extracted from YYH and BJT played a vital role in the treatment of asthma. Cytokines (IL6, TNF, and IL1B) and chemokines (CXCL8 and CCL2) could be recognized as hub genes. The cytokine-mediated signaling pathway (IL-17 signaling pathway) was the main mechanism for CKZI in the treatment of asthma. However, further *in vitro* and *in vivo* experiments needed to be established to verify the results analyzed by the network pharmacology method.

## Data Availability

Data are obtained through the authoritative database and software analysis.

## Abbreviations

CKZI: Chuankezhi injection
TCMSP: Traditional Chinese Medicine Systems Pharmacology Database and Analysis Platform
CNKI: China National Knowledge Infrastructure Website
OMIM: Online Mendelian Inheritance in Man
PPI: Protein-protein interaction
GO: Gene Ontology
KEGG: Kyoto Encyclopedia of Genes and Genomes
BP: Biological process
MF: Molecular function
CC: Cellular component
ICS: Inhaled corticosteroid
LABA: Long-acting *β*-receptor agonist
SABA: Inhaled or nebulized short-acting *β*2-agonist
TCM: Traditional Chinese medicine
YYH: Yingyanghuo
BJT: Bajitian
DC-T-P: Disease-related candidate compound-target-pathway
DL: Drug-likeness
OB: Oral bioavailability
ADME: Absorption, distribution, metabolism, and excretion
TNF-*α*: Tumor necrosis factor-*α*.

## Supplementary Materials

Supplementary Materials Table S1: chemical profiles and structure candidate compounds; Table S2: degree values of candidate compounds in the herb-candidate compound-target network; Table S3: degree values of potential targets in the herb-candidate compound-target network; Table S4: degree values of gene symbols in the protein-protein interaction network; Table S5: degree values of compounds in the disease-related candidate compound-target-pathway network; Table S6: degree values of gene symbols in the disease-related candidate compound-target-pathway network; Table S7: degree values of pathways in the disease-related candidate compound-target-pathway network.
