# Supplementary material for: Investigation of the Mechanisms of Chuankezhi Injection in the Treatment of Asthma Based on the Network Pharmacology Approach
Source: Evid Based Complement Alternat Med. 2021 Jun 10;2021:5517041. doi: 10.1155/2021/5517041 (PMC8213469; doi:10.1155/2021/5517041)
Supplement: Supplementary Materials — Table S1: chemical profiles and structure candidate compounds; Table S2: degree values of candidate compounds in the herb-candidate compound-target network; Table S3: degree values of potential targets in the herb-candidate compound-target network; Table S4: degree values of gene symbols in the protein-protein interaction network; Table S5: degree values of compounds in the disease-related candidate compound-target-pathway network; Table S6: degree values of gene symbols in the disease-related candidate compound-target-pathway network; Table S7: degree values of pathways in the disease-related candidate compound-target-pathway network. [file 5517041.f1.pdf]

Supplementary Information

Hao Zhu[1], Yuhuan Shi[1], Shanshan Jiang[1], Xiuxiu Jiao[1], Hui Zhu[1], Rong Wang[1], Yongfang Yuan[*1]

[1] Department of Pharmacy, Shanghai Ninth People's Hospital, Shanghai Jiao Tong University School of Medicine, Shanghai, China.

*Corresponding author: Yongfang Yuan, Department of Pharmacy, Shanghai Ninth People's Hospital, Shanghai Jiao Tong University School of Medicine, 639 Zhi Zao Ju Road, Shanghai 200011, China. Email: nmxyyf@126.com

Table S1 Chemical profiles and structure candidate compounds

Abbreviation	Mol ID	Name	Molecular formula	CAS No.	MW (g/mol)	OB (%)	DL	Chemical structure
YYH1	MOL000006	luteolin	$C_{15}H_{10}O_6$	491-70-3	286.25	36.16	0.25	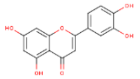
YYH2	MOL000098	quercetin	$C_{15}H_{10}O_7$	117-39-5	302.25	46.43	0.28	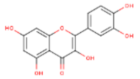

A	MOL000359	3-epi-beta-sitosterol	$C_{29}H_{50}O$	N/A	414.79	36.91	0.75	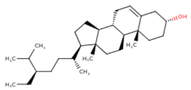
YYH3	MOL000422	kaempferol	$C_{15}H_{10}O_6$	520-18-3	286.25	41.88	0.24	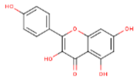
YYH4	MOL000622	magnograndiolide	$C_{15}H_{22}O_4$	92618-98-9	266.37	63.71	0.19	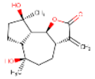
YYH5	MOL001510	24-epicampesterol	$C_{28}H_{48}O$	4651-51-8	400.76	37.58	0.71	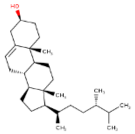
YYH6	MOL001645	linoleyl acetate	$C_{20}H_{36}O_2$	5999-95-1	308.56	42.1	0.2	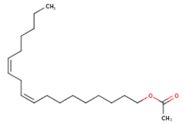
YYH7	MOL001771	clionasterol	$C_{29}H_{50}O$	83-47-6	414.79	36.91	0.75	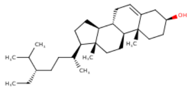

YYH8	MOL001792	liquiritigenin	$C_{15}H_{12}O_4$	578-86-9	256.27	32.76	0.18	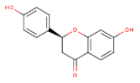
YYH9	MOL003044	chryseriol	$C_{16}H_{12}O_6$	491-71-4	300.28	35.85	0.27	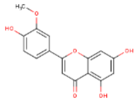
YYH10	MOL003542	8-isopentenyl-kaempferol	$C_{20}H_{18}O_6$	28610-31-3	354.38	38.04	0.39	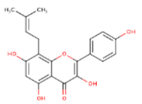
YYH11	MOL004372	sagittatoside A	$C_{33}H_{40}O_{15}$	118525-35-2	676.73	8.5	0.57	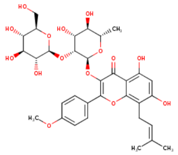
YYH12	MOL004373	anhydroicaritin	$C_{21}H_{20}O_6$	118525-40-9	368.41	45.41	0.44	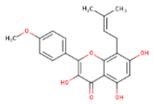
YYH13	MOL004374	sagittatoside B	$C_{32}H_{38}O_{14}$	118525-36-3	646.7	5.58	0.64	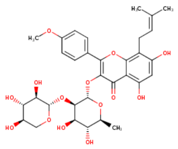

YYH14	MOL004380	2,7-dihydrohomoerysotrine	$C_{20}H_{27}NO_3$	51095-85-3	329.48	39.14	0.49	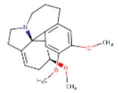
YYH15	MOL004382	yinyanghuo A	$C_{25}H_{24}O_6$	174391-72-1	420.49	56.96	0.77	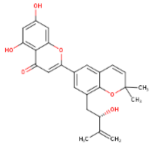
YYH16	MOL004384	yinyanghuo C	$C_{20}H_{16}O_5$	149182-47-8	336.36	45.67	0.5	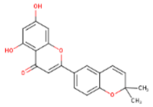
YYH17	MOL004386	yinyanghuo E	$C_{20}H_{16}O_6$	174286-26-1	352.36	51.63	0.55	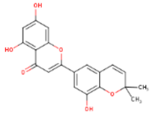
YYH18	MOL004388	6-hydroxy-11,12-dimethoxy-2,2-dimethyl-1,8-dioxo-2,3,4,8-tetrahydro-1H-isochromeno[3,4-h]isoquinolin-2-ium	$C_{20}H_{20}NO_6^+$	N/A	370.41	60.64	0.66	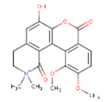
YYH19	MOL004391	8-prenyl-flavone	$C_{20}H_{18}O_2$	N/A	290.38	48.54	0.25	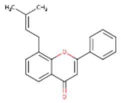

YYH20	MOL004396	1,2-bis(4-hydroxy-3-methoxyphenyl)propan-1,3-diol	$C_{17}H_{20}O_6$	N/A	320.37	52.31	0.22	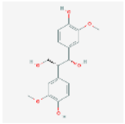
YYH21	MOL004425	icariin	$C_{33}H_{40}O_{15}$	489-32-7	676.73	41.58	0.61	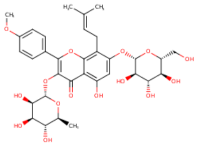
YYH22	MOL004427	icariside A7	$C_{23}H_{26}O_{10}$	N/A	462.49	31.91	0.86	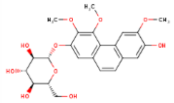
YYH23	MOL004430	icariside I	$C_{27}H_{30}O_{11}$	56725-99-6	530.57	21.88	0.85	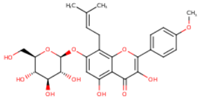
YYH24	MOL004431	icariside II	$C_{27}H_{30}O_{10}$	113558-15-9	514.57	3.7	0.84	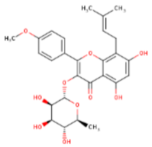
YYH25	N/A	chlorogenic acid	$C_{16}H_{18}O_9$	327-97-9	354.31	N/A	N/A	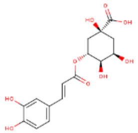

BJT1	MOL000358	beta-sitosterol	$C_{29}H_{50}O$	83-46-5	414.79	36.91	0.75	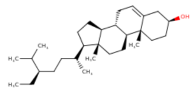
A	MOL000359	3-epi-beta-sitosterol	$C_{29}H_{50}O$	N/A	414.79	36.91	0.75	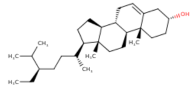
BJT2	MOL001649	2-hydroxy-3-methylanthraquinone	$C_{15}H_{10}O_3$	17241-40-6	238.25	26.09	0.18	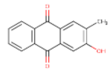
BJT3	MOL002879	diop	$C_{24}H_{38}O_4$	27554-26-3	390.62	43.59	0.39	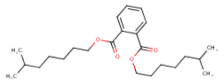
BJT4	MOL002883	ethyl oleate	$C_{20}H_{38}O_2$	111-62-6	310.58	32.4	0.19	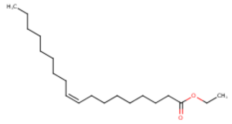

BJT5	MOL006147	alizarin-2-methylether	$C_{15}H_{10}O_4$	6003-11-8	254.25	32.81	0.21	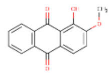
BJT6	MOL009495	1,5,15-tri-O-methylmorindol	$C_{18}H_{16}O_6$	942609-65-6	328.34	95.85	0.37	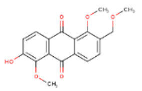
BJT7	MOL009496	1,3,5-trihydroxy-2-methoxy-6-(methoxymethyl)anthracene-9,10-dione	$C_{17}H_{14}O_7$	N/A	330.31	80.42	0.38	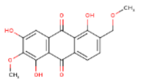
BJT8	MOL009498	1,6-Dihydroxy-2,4-dimethoxyanthraquinone	$C_{16}H_{12}O_6$	142878-33-9	300.28	18.42	0.3	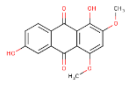
BJT9	MOL009500	9,10-anthracenedione	$C_{17}H_{14}O_6$	877238-55-6	314.31	104.54	0.34	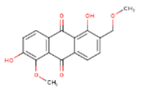
BJT10	MOL009504	1-hydroxy-6-hydroxymethyl anthracenequinone	$C_{15}H_{10}O_4$	N/A	254.25	81.77	0.21	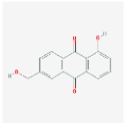
BJT11	MOL009514	1-methoxy-2-hydroxyanthraquinone	$C_{15}H_{10}O_4$	6170-6-5	254.25	8.7	0.21	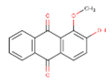

BJT12	MOL009519	(2R,3S)-(+)-3',5-Dihydroxy-4 ,7-dimethoxydihydroflavonol	$C_{17}H_{16}O_7$	N/A	332.33	77.24	0.33	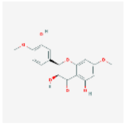
BJT13	MOL009524	3beta,20(R),5-alkenyl-stigmastol	$C_{29}H_{50}O$	N/A	414.79	36.91	0.75	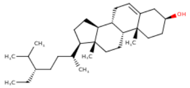
BJT14	MOL009537	americanin A	$C_{18}H_{16}O_6$	69506-79-2	328.34	46.71	0.35	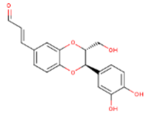
BJT15	MOL009547	deacetyl asperulosidic acid	$C_{16}H_{22}O_{11}$	14259-55-3	390.38	16.9	0.45	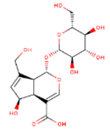
BJT16	MOL009551	isoprincepin	$C_{27}H_{26}O_9$	N/A	494.53	49.12	0.77	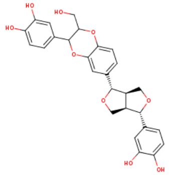
BJT17	MOL009553	monotropein	$C_{16}H_{22}O_{11}$	5945-50-6	390.38	2.53	0.44	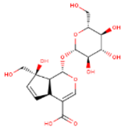

BJT18	MOL009562	ohioensin-A	$C_{23}H_{16}O_5$	121353-47-7	372.39	38.13	0.76	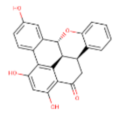

Table S2 Degree values of candidate compounds in herb-candidate compound-target network

Candidate Compounds			
**Molecule ID**	**Molecule Name**	**Abbreviation**	**Degree**
MOL000098	quercetin	YYH2	205
MOL000006	luteolin	YYH1	83
MOL000422	kaempferol	YYH3	75
MOL004373	anhydroicaritin	YYH12	39
MOL004380	2,7-dihydrohomoerysotrine	YYH14	39
MOL000358	beta-sitosterol	BJT1	38
MOL001649	2-hydroxy-3-methylanthraquinone	BJT2	34
MOL004391	8-prenyl-flavone	YYH19	31
MOL003542	8-isopentenyl-kaempferol	YYH10	30
MOL003044	chryseriol	YYH9	21
MOL004372	sagittatoside A	YYH11	18

MOL009514	1-methoxy-2-hydroxyanthraquinone	BJT11	18
MOL004430	icariside I	YYH23	17
MOL009495	1,5,15-tri-O-methylmorindol	BJT6	15
MOL006147	alizarin-2-methylether	BJT5	14
MOL001792	liquiritigenin	YYH8	13
MOL009500	9,10-anthracenedione	BJT9	13
MOL009504	1-hydroxy-6-hydroxymethyl anthracenequinone	BJT10	13
MOL004384	yinyanghuo C	YYH16	12
MOL004386	yinyanghuo E	YYH17	12
MOL004396	1,2-bis(4-hydroxy-3-methoxyphenyl)propan-1,3-diol	YYH20	12
MOL009537	americanin A	BJT14	12
MOL009496	1,3,5-trihydroxy-2-methoxy-6-(methoxymethyl)anthracene-9,10-dione	BJT7	11
MOL004382	yinyanghuo A	YYH15	10
MOL009519	(2R,3S)-(+)-3',5-dihydroxy-4 ,7-	BJT12	10

	dimethoxydihydroflavonol		
MOL004374	sagittatoside B	YYH13	8
MOL000359	3-epi-beta-sitosterol	A	8
MOL004388	6-hydroxy-11,12-dimethoxy-2,2-dimethyl-1,8-dioxo-2,3,4,8-tetrahydro-1H-isochromeno[3,4-h]isoquinolin-2-ium	YYH18	7
MOL009498	1,6-Dihydroxy-2,4-dimethoxyanthraquinone	BJT8	7
MOL000622	magnograndiolide	YYH4	5
MOL001645	linoleyl acetate	YYH6	5
MOL004427	icariside A7	YYH22	4
MOL002879	diop	BJT3	4
MOL009562	ohioensin-A	BJT18	4
MOL001510	24-epicampesterol	YYH5	3
MOL001771	clionasterol	YYH7	3
MOL004425	icariin	YYH21	3

MOL004431	icariside II	YYH24	3
N/A	chlorogenic acid	YYH25	3
MOL009551	isoprincepin	BJT16	3
MOL009553	monotropein	BJT17	3
MOL002883	ethyl oleate	BJT4	2
MOL009524	3beta,20(R),5-alkenyl-stigmastol	BJT13	2
MOL009547	deacetyl asperulosidic acid	BJT15	2

Table S3 Degree values of potential targets in herb-candidate compound-target network

**Potential Targets**		
**Gene symbol**	**Target name**	**Degree**
PTGS2	Prostaglandin G/H synthase 2	30
NCOA2	Nuclear receptor coactivator 2	28
HSP90AA1	Heat shock protein HSP 90-alpha	23
PTGS1	Prostaglandin G/H synthase 1	22
TOP2A	DNA topoisomerase 2-alpha	18
SCN5A	Sodium channel protein type 5 subunit alpha	15

PIK3CG	Phosphatidylinositol 4,5-bisphosphate 3-kinase catalytic subunit gamma isoform	14
CALM1	Calmodulin-1	14
PRSS1	Trypsin-1	14
F10	Coagulation factor X	13
AR	Androgen receptor	13
PRKACA	cAMP-dependent protein kinase catalytic subunit alpha	12
DPP4	Dipeptidyl peptidase 4	12
GABRA1	Gamma-aminobutyric acid receptor subunit alpha-1	11
RXRA	Retinoic acid receptor RXR-alpha	11
ADRB2	Beta-2 adrenergic receptor	10
ESR1	Estrogen receptor	10
F7	Coagulation factor VII	10
KCNH2	Potassium voltage-gated channel subfamily H member 2	8
PDE3A	cGMP-inhibited 3',5'-cyclic phosphodiesterase A	8
CDK2	Cyclin-dependent kinase 2	8

PIM1	Serine/threonine-protein kinase pim-1	8
PPARG	Peroxisome proliferator-activated receptor gamma	8
PGR	Progesterone receptor	7
SLC6A4	Sodium-dependent serotonin transporter	7
GSK3B	Glycogen synthase kinase-3 beta	7
ACHE	Acetylcholinesterase	7
F2R	Proteinase-activated receptor 1	7
NOS2	Nitric oxide synthase, inducible	7
CHRM3	Muscarinic acetylcholine receptor M3	6
CHRM1	Muscarinic acetylcholine receptor M1	6
ADRA1B	Alpha-1B adrenergic receptor	6
CHRNA7	Neuronal acetylcholine receptor subunit alpha-7	6
CASP3	Caspase-3	6
IGHG1	Immunoglobulin heavy constant gamma 1	6
NCOA1	Nuclear receptor coactivator 1	6
PKIA	cAMP-dependent protein kinase inhibitor alpha	5
CCNA2	Cyclin-A2	5

CA2	Carbonic anhydrase 2	5
KDR	Vascular endothelial growth factor receptor 2	5
GABRA2	Gamma-aminobutyric acid receptor subunit alpha-2	4
CHRM2	Muscarinic acetylcholine receptor M2	4
JUN	Transcription factor AP-1	4
SLC6A3	Sodium-dependent dopamine transporter	4
MAOB	Amine oxidase	4
KCNMA1	Calcium-activated potassium channel subunit alpha-1	4
AKR1B1	Aldo-keto reductase family 1 member B1	4
CYP1B1	Cytochrome P450 1B1	4
NOS3	Nitric oxide synthase, endothelial	4
MAPK14	Mitogen-activated protein kinase 14	4
CHEK1	Serine/threonine-protein kinase Chk1	4
DRD1	D(1A) dopamine receptor	3
CHRM4	Muscarinic acetylcholine receptor M4	3
HTR2A	5-hydroxytryptamine receptor 2A	3
ADRA1A	Alpha-1A adrenergic receptor	3

CHRNA2	Neuronal acetylcholine receptor subunit alpha-2	3
OPRM1	Mu-type opioid receptor	3
BCL2	Apoptosis regulator Bcl-2	3
BAX	Apoptosis regulator BAX	3
CASP9	Caspase-9	3
CHRM5	Muscarinic acetylcholine receptor M5	3
RELA	Transcription factor p65	3
AKT1	RAC-alpha serine/threonine-protein kinase	3
TNF	Tumor necrosis factor	3
XDH	Xanthine dehydrogenase/oxidase	3
MMP1	Interstitial collagenase	3
HMOX1	Heme oxygenase 1	3
ICAM1	Intercellular adhesion molecule 1	3
GSTP1	Glutathione S-transferase P	3
SLC2A4	Solute carrier family 2, facilitated glucose transporter member 4	3
INSR	Insulin receptor	3

NOX4	NADPH oxidase 4	3
MAOA	Amine oxidase	3
FLT3	Receptor-type tyrosine-protein kinase FLT3	3
ALOX5	Arachidonate 5-lipoxygenase	3
CA7	Carbonic anhydrase 7	3
CA12	Carbonic anhydrase 12	3
ABCG2	Broad substrate specificity ATP-binding cassette transporter ABCG2	3
ABCC1	Multidrug resistance-associated protein 1	3
SLC6A2	Sodium-dependent noradrenaline transporter	3
ESR2	Estrogen receptor beta	3
PDE5A	cGMP-specific 3',5'-cyclic phosphodiesterase	3
GABRA3	Gamma-aminobutyric acid receptor subunit alpha-3	2
CASP8	Caspase-8	2
PRKCA	Protein kinase C alpha type	2
TGFB1	Transforming growth factor beta-1 proprotein	2
PON1	Serum paraoxonase/arylesterase 1	2

NR3C2	Mineralocorticoid receptor	2
OPRD1	Delta-type opioid receptor	2
HRH1	Histamine H1 receptor	2
HTR2C	5-hydroxytryptamine receptor 2C	2
ADRA2B	Alpha-2B adrenergic receptor	2
DRD2	D(2) dopamine receptor	2
LTA4H	Leukotriene A-4 hydrolase	2
EGFR	Epidermal growth factor receptor	2
VEGFA	Vascular endothelial growth factor A	2
CCND1	G1/S-specific cyclin-D1	2
BCL2L1	Bcl-2-like protein 1	2
CDKN1A	Cyclin-dependent kinase inhibitor 1	2
MMP2	72 kDa type IV collagenase	2
MMP9	Matrix metalloproteinase-9	2
MAPK1	Mitogen-activated protein kinase 1	2
IL10	Interleukin-10	2
RB1	Retinoblastoma-associated protein	2

IL6	Interleukin-6	2
TP53	Cellular tumor antigen p53	2
NFKBIA	NF-kappa-B inhibitor alpha	2
TOP1	DNA topoisomerase 1	2
ERBB2	Receptor tyrosine-protein kinase erbB-2	2
BIRC5	Baculoviral IAP repeat-containing protein 5	2
IL2	Interleukin-2	2
CCNB1	G2/mitotic-specific cyclin-B1	2
TYR	Tyrosinase	2
IFNG	Interferon gamma	2
CD40LG	CD40 ligand	2
MET	Hepatocyte growth factor receptor	2
ADORA1	Adenosine receptor A1	2
GLO1	Lactoylglutathione lyase	2
PARP1	Poly [ADP-ribose] polymerase 1	2
CA4	Carbonic anhydrase 4	2
AKR1B10	Aldo-keto reductase family 1 member B10	2

AHSA1	Activator of 90 kDa heat shock protein ATPase homolog 1	2
STAT1	Signal transducer and activator of transcription 1-alpha/beta	2
CDK1	Cyclin-dependent kinase 1	2
CYP3A4	Cytochrome P450 3A4	2
CYP1A2	Cytochrome P450 1A2	2
CYP1A1	Cytochrome P450 1A1	2
SELE	E-selectin	2
VCAM1	Vascular cell adhesion protein 1	2
NR1I2	Nuclear receptor subfamily 1 group I member 2	2
HAS2	Hyaluronan synthase 2	2
AHR	Aryl hydrocarbon receptor	2
PSMD3	26S proteasome non-ATPase regulatory subunit 3	2
NR1I3	Nuclear receptor subfamily 1 group I member 3	2
DIO1	Type I iodothyronine deiodinase	2
GSTM1	Glutathione S-transferase Mu 1	2

GSTM2	Glutathione S-transferase Mu 2	2
CYP19A1	Aromatase	2
HSD17B2	Estradiol 17-beta-dehydrogenase 2	2
ABCB1	ATP-dependent translocase ABCB1	2
AKR1C3	Aldo-keto reductase family 1 member C3	2
ADRB1	Beta-1 adrenergic receptor	2
ADRA1D	Alpha-1D adrenergic receptor	2
GABRA5	Gamma-aminobutyric acid receptor subunit alpha-5	1
MAP2	Microtubule-associated protein 2	1
CDK4	Cyclin-dependent kinase 4	1
MDM2	E3 ubiquitin-protein ligase Mdm2	1
APP	Amyloid-beta precursor protein	1
PCNA	Proliferating cell nuclear antigen	1
CASP7	Caspase-7	1
MCL1	Induced myeloid leukemia cell differentiation protein Mcl-1	1
IL4	Interleukin-4	1

XIAP	E3 ubiquitin-protein ligase XIAP	1
PTGES	Prostaglandin E synthase	1
NUF2	Kinetochore protein Nuf2	1
ADCY2	Adenylate cyclase type 2	1
CDK5R1	Cyclin-dependent kinase 5 activator 1	1
CCNB3	G2/mitotic-specific cyclin-B3	1
SYK	Tyrosine-protein kinase SYK	1
TTR	Transthyretin	1
MMP12	Macrophage metalloelastase	1
CD38	ADP-ribosyl cyclase/cyclic ADP-ribose hydrolase 1	1
TNKS2	Poly [ADP-ribose] polymerase tankyrase-2	1
TNKS	Poly [ADP-ribose] polymerase tankyrase-1	1
ARG1	Arginase-1	1
MMP3	Stromelysin-1	1
FOS	Proto-oncogene c-Fos	1
EIF6	Eukaryotic translation initiation factor 6	1
PLAU	Urokinase-type plasminogen activator	1

EGF	Pro-epidermal growth factor	1
ELK1	ETS domain-containing protein Elk-1	1
POR	NADPH--cytochrome P450 reductase	1
ODC1	Ornithine decarboxylase	1
RAF1	RAF proto-oncogene serine/threonine-protein kinase	1
SOD1	Superoxide dismutase	1
HIF1A	Hypoxia-inducible factor 1-alpha	1
RUNX1T1	Protein CBFA2T1	1
HERC5	E3 ISG15--protein ligase HERC5	1
HSPA5	Endoplasmic reticulum chaperone BiP	1
ACACA	Acetyl-CoA carboxylase 1	1
CAV1	Caveolin-1	1
MYC	Myc proto-oncogene protein	1
F3	Tissue factor	1
GJA1	Gap junction alpha-1 protein	1
IL1B	Interleukin-1 beta	1
CCL2	C-C motif chemokine 2	1

PTGER3	Prostaglandin E2 receptor EP3 subtype	1
CXCL8	Interleukin-8	1
PRKCB	Protein kinase C beta type	1
DUOX2	Dual oxidase 2	1
HSPB1	Heat shock protein beta-1	1
SULT1E1	Sulfotransferase 1E1	1
MGAM	Maltase-glucoamylase, intestinal	1
PLAT	Tissue-type plasminogen activator	1
THBD	Thrombomodulin	1
SERPINE1	Plasminogen activator inhibitor 1	1
COL1A1	Collagen alpha-1	1
PTEN	Phosphatidylinositol 3,4,5-trisphosphate 3-phosphatase and dual-specificity protein phosphatase PTEN	1
IL1A	Interleukin-1 alpha	1
MPO	Myeloperoxidase	1
NCF1	Neutrophil cytosol factor 1	1
NFE2L2	Nuclear factor erythroid 2-related factor 2	1

NQO1	NAD(P)H dehydrogenase [quinone] 1	1
COL3A1	Collagen alpha-1	1
CXCL11	C-X-C motif chemokine 11	1
CXCL2	C-X-C motif chemokine 2	1
DCAF5	DDB1- and CUL4-associated factor 5	1
CHEK2	Serine/threonine-protein kinase Chk2	1
CLDN4	Claudin-4	1
PPARA	Peroxisome proliferator-activated receptor alpha	1
PPARD	Peroxisome proliferator-activated receptor delta	1
HSF1	Heat shock factor protein 1	1
CRP	C-reactive protein	1
CXCL10	C-X-C motif chemokine 10	1
CHUK	Inhibitor of nuclear factor kappa-B kinase subunit alpha	1
SPP1	Osteopontin	1
RUNX2	Runt-related transcription factor 2	1
RASSF1	Ras association domain-containing protein 1	1

E2F1	Transcription factor E2F1	1
E2F2	Transcription factor E2F2	1
ACP3	Prostatic acid phosphatase	1
CTSD	Cathepsin D	1
IGFBP3	Insulin-like growth factor-binding protein 3	1
IGF2	Insulin-like growth factor II	1
IRF1	Interferon regulatory factor 1	1
ERBB3	Receptor tyrosine-protein kinase erbB-3	1
PCOLCE	Procollagen C-endopeptidase enhancer 1	1
NPEPPS	Puromycin-sensitive aminopeptidase	1
HK2	Hexokinase-2	1
NKX3-1	Homeobox protein Nkx-3.1	1
RASA1	Ras GTPase-activating protein 1	1
AVPR2	Vasopressin V2 receptor	1
IGF1R	Insulin-like growth factor 1 receptor	1
F2	Prothrombin	1
AURKB	Aurora kinase B	1

DRD4	D(4) dopamine receptor	1
PIK3R1	Phosphatidylinositol 3-kinase regulatory subunit alpha	1
ADORA2A	Adenosine receptor A2a	1
DAPK1	Death-associated protein kinase 1	1
PYGL	Glycogen phosphorylase, liver form	1
CA1	Carbonic anhydrase 1	1
SRC	Proto-oncogene tyrosine-protein kinase Src	1
PTK2	Focal adhesion kinase 1	1
MMP13	Collagenase 3	1
CA3	Carbonic anhydrase 3	1
ALOX15	Arachidonate 15-lipoxygenase	1
PLK1	Serine/threonine-protein kinase PLK1	1
CA6	Carbonic anhydrase 6	1
PKN1	Serine/threonine-protein kinase N1	1
CA14	Carbonic anhydrase 14	1
CA9	Carbonic anhydrase 9	1
CSNK2A1	Casein kinase II subunit alpha	1

ALOX12	Arachidonate 12-lipoxygenase, 12S-type	1
NEK2	Serine/threonine-protein kinase Nek2	1
CXCR1	C-X-C chemokine receptor type 1	1
CAMK2B	Calcium/calmodulin-dependent protein kinase type II subunit beta	1
ALK	ALK tyrosine kinase receptor	1
NEK6	Serine/threonine-protein kinase Nek6	1
PLA2G1B	Phospholipase A2	1
CA5A	Carbonic anhydrase 5A, mitochondrial	1
BACE1	Beta-secretase 1	1
AXL	Tyrosine-protein kinase receptor UFO	1
NUAK1	NUAK family SNF1-like kinase 1	1
AKR1C2	Aldo-keto reductase family 1 member C2	1
AKR1C1	Aldo-keto reductase family 1 member C1	1
AKR1C4	Aldo-keto reductase family 1 member C4	1
CA13	Carbonic anhydrase 13	1
AKR1A1	Aldo-keto reductase family 1 member A1	1

GPR35	G-protein coupled receptor 35	1
IKBKB	Inhibitor of nuclear factor kappa-B kinase subunit beta	1
MAPK8	Mitogen-activated protein kinase 8	1
PPP3CA	Serine/threonine-protein phosphatase 2B catalytic subunit alpha isoform	1
SLPI	Antileukoproteinase	1
HSD17B1	Estradiol 17-beta-dehydrogenase 1	1
ESRRA	Steroid hormone receptor ERR1	1
GRIA2	Glutamate receptor 2	1
GABRA6	Gamma-aminobutyric acid receptor subunit alpha-6	1
PYGM	Glycogen phosphorylase, muscle form	1
RXRB	Retinoic acid receptor RXR-beta	1
ADH1C	Alcohol dehydrogenase 1C	1
PRSS3	Trypsin-3	1
DRD5	D(1B) dopamine receptor	1
ADRA2A	Alpha-2A adrenergic receptor	1
HTR1A	5-hydroxytryptamine receptor 1A	1

HTR3A	5-hydroxytryptamine receptor 3A	1
HTR1B	5-hydroxytryptamine receptor 1B	1
GABRG3	Gamma-aminobutyric acid receptor subunit gamma-3	1
GABRE	Gamma-aminobutyric acid receptor subunit epsilon	1

Table S4 Degree values of gene symbols in protein-protein interaction network

**Gene Symbol**	**Target Name**	**Degree**
IL6	Interleukin-6	40
TNF	Tumor necrosis factor	38
IL1B	Interleukin-1 beta	35
CXCL8	Interleukin-8	32
CCL2	C-C motif chemokine 2	29
IL10	Interleukin-10	29
VEGFA	Vascular endothelial growth factor A	28
JUN	Transcription factor AP-1	28
PTGS2	Prostaglandin G/H synthase 2	27
ICAM1	Intercellular adhesion molecule 1	27

MMP9	Matrix metalloproteinase-9	27
IL4	Interleukin-4	24
MAPK1	Mitogen-activated protein kinase 1	24
CRP	C-reactive protein	20
EGF	Pro-epidermal growth factor	20
IL2	Interleukin-2	19
MMP2	72 kDa type IV collagenase	19
IFNG	Interferon gamma	19
STAT1	Signal transducer and activator of transcription 1-alpha/beta	19
CXCL10	C-X-C motif chemokine 10	18
MMP1	Interstitial collagenase	17
HMOX1	Heme oxygenase 1	17
NOS3	Nitric oxide synthase, endothelial	17
VCAM1	Vascular cell adhesion protein 1	17
SELE	E-selectin	16
TGFB1	Transforming growth factor beta-1 proprotein	16

SERPINE1	Plasminogen activator inhibitor 1	16
MAPK14	Mitogen-activated protein kinase 14	15
IL1A	Interleukin-1 alpha	15
PPARG	Peroxisome proliferator-activated receptor gamma	14
F2	Prothrombin	13
MPO	Myeloperoxidase	12
CD40LG	CD40 ligand	12
MMP3	Stromelysin-1	11
NOS2	Nitric oxide synthase, inducible	11
F3	Tissue factor	11
IRF1	Interferon regulatory factor 1	10
F2R	Proteinase-activated receptor 1	10
ARG1	Arginase-1	9
CHRM2	Muscarinic acetylcholine receptor M2	8
CXCR1	C-X-C chemokine receptor type 1	7
THBD	Thrombomodulin	7
ADRB2	Beta-2 adrenergic receptor	6

IGFBP3	Insulin-like growth factor-binding protein 3	6
ADRA1B	Alpha-1B adrenergic receptor	5
OPRM1	Mu-type opioid receptor	5
ADORA1	Adenosine receptor A1	5
COL1A1	Collagen alpha-1	5
ALOX15	Arachidonate 15-lipoxygenase	5
PTGS1	Prostaglandin G/H synthase 1	5
ALOX5	Arachidonate 5-lipoxygenase	5
CASP8	Caspase-8	5
PLAU	Urokinase-type plasminogen activator	5
CYP3A4	Cytochrome P450 3A4	4
CHRM3	Muscarinic acetylcholine receptor M3	4
HRH1	Histamine H1 receptor	4
SYK	Tyrosine-protein kinase SYK	4
GSTP1	Glutathione S-transferase P	3
SOD1	Superoxide dismutase	2
GSTM1	Glutathione S-transferase Mu 1	2

PIK3CG	Phosphatidylinositol 4,5-bisphosphate 3-kinase catalytic subunit gamma isoform	2
LTA4H	Leukotriene A-4 hydrolase	2
MMP12	Macrophage metalloelastase	1
ABCB1	ATP-dependent translocase ABCB1	1
ADRB1	Beta-1 adrenergic receptor	1

Table S5 Degree values of compounds in disease related candidate compound-target-pathway network

Compound	Name	Degree
YYH2	quercetin	49
YYH1	luteolin	23
YYH3	kaempferol	21
YYH19	8-prenyl-flavone	13
BJT1	beta-sitosterol	13
YYH14	2,7-dihydrohomoerysotrine	11
YYH12	anhydroicaritin	11
BJT2	2-hydroxy-3-methylanthraquinone	10
YYH9	chryseriol	7
YYH10	8-isopentenyl-kaempferol	6
YYH8	liquiritigenin	6
BJT11	1-methoxy-2-hydroxyanthraquinone	5
BJT10	1-hydroxy-6-hydroxymethyl anthracenequinone	5
BJT5	alizarin-2-methylether	5
YYH23	icariside I	4

YYH20	1,2-bis(4-hydroxy-3-methoxyphenyl)propan-1,3-diol	4
YYH11	sagittatoside A	4
BJT12	(2R,3S)-(+)-3',5-dihydroxy-4 ,7-dimethoxydihydroflavonol	4
BJT9	9,10-anthracenedione	4
BJT8	1,6-dihydroxy-2,4-dimethoxyanthraquinone	4
BJT6	1,5,15-tri-O-methylmorindol	4
YYH18	6-hydroxy-11,12-dimethoxy-2,2-dimethyl-1,8-dioxo-2,3,4,8-tetrahydro-1H-isochromeno[3,4-h]isoquinolin-2-ium	3
YYH17	yinyanghuo E	3
YYH16	yinyanghuo C	3
YYH6	linoleyl acetate	3
BJT18	ohioensin-A	3
BJT7	1,3,5-trihydroxy-2-methoxy-6-(methoxymethyl)anthracene-9,10-dione	3

BJT3	diop	3
YYH22	icariside A7	2
YYH15	yinyanghuo A	2
BJT14	americanin A	2

Table S6 Degree values of gene symbols in disease related candidate compound-target-pathway network

**Gene Symbol**	**Target Name**	**Degree**
PTGS2	Prostaglandin G/H synthase 2	34
PTGS1	Prostaglandin G/H synthase 1	23
PIK3CG	Phosphatidylinositol 4,5-bisphosphate 3-kinase catalytic subunit gamma isoform	15
MAPK1	Mitogen-activated protein kinase 1	13
F2R	Proteinase-activated receptor 1	11
TNF	Tumor necrosis factor	11
ADRB2	Beta-2 adrenergic receptor	11
MAPK14	Mitogen-activated protein kinase 14	10
NOS2	Nitric oxide synthase, inducible	10

PPARG	Peroxisome proliferator-activated receptor gamma	10
IL6	Interleukin-6	10
JUN	Transcription factor AP-1	10
NOS3	Nitric oxide synthase, endothelial	9
CXCL8	Interleukin-8	9
SLC6A4	Sodium-dependent serotonin transporter	8
STAT1	Signal transducer and activator of transcription 1-alpha/beta	7
ICAM1	Intercellular adhesion molecule 1	7
MMP9	Matrix metalloproteinase-9	7
CHRM3	Muscarinic acetylcholine receptor M3	7
ADRA1B	Alpha-1B adrenergic receptor	7
CASP8	Caspase-8	7
VCAM1	Vascular cell adhesion protein 1	6
MMP2	72 kDa type IV collagenase	6
VEGFA	Vascular endothelial growth factor A	6

CHRM2	Muscarinic acetylcholine receptor M2	6
TGFB1	Transforming growth factor beta-1 proprotein	6
EGF	Pro-epidermal growth factor	5
SERPINE1	Plasminogen activator inhibitor 1	5
IL1B	Interleukin-1 beta	5
ALOX5	Arachidonate 5-lipoxygenase	5
GSTP1	Glutathione S-transferase P	5
IL4	Interleukin-4	5
IFNG	Interferon gamma	5
IL2	Interleukin-2	5
MMP1	Interstitial collagenase	5
PLAU	Urokinase-type plasminogen activator	4
CXCL10	C-X-C motif chemokine 10	4
CCL2	C-C motif chemokine 2	4
SYK	Tyrosine-protein kinase SYK	4
HMOX1	Heme oxygenase 1	4
OPRM1	Mu-type opioid receptor	4

ADRB1	Beta-1 adrenergic receptor	3
GSTM1	Glutathione S-transferase Mu 1	3
IGFBP3	Insulin-like growth factor-binding protein 3	3
MMP3	Stromelysin-1	3
THBD	Thrombomodulin	3
SELE	E-selectin	3
IL1A	Interleukin-1 alpha	3
F3	Tissue factor	3
COL1A1	Collagen alpha-1	3
CD40LG	CD40 ligand	3
IL10	Interleukin-10	3
HRH1	Histamine H1 receptor	3
CXCR1	C-X-C chemokine receptor type 1	2
ALOX15	Arachidonate 15-lipoxygenase	2
F2	Prothrombin	2
IRF1	Interferon regulatory factor 1	2
MPO	Myeloperoxidase	2

ADORA2A	Adenosine receptor A2a	2

Table S7 Degree values of pathways in disease related candidate compound-target-pathway network

Pathway	Degree
AGE-RAGE signaling pathway in diabetic complications	21
Pathways in cancer	17
IL-17 signaling pathway	16
PI3K-Akt signaling pathway	12
Inflammatory bowel disease (IBD)	11
Calcium signaling pathway	10
HIF-1 signaling pathway	9
NF-kappa B signaling pathway	9
Transcriptional misregulation in cancer	8
HTLV-I infection	7
Hepatitis C	7
Chemokine signaling pathway	6

Serotonergic synapse	6
Estrogen signaling pathway	6
Complement and coagulation cascades	6
Fc epsilon RI signaling pathway	6
Leukocyte transendothelial migration	5
RIG-I-like receptor signaling pathway	5
Platinum drug resistance	4
p53 signaling pathway	3
